# What explains rare and conspicuous colours in a snail? A test of time-series data against models of drift, migration or selection

**DOI:** 10.1038/hdy.2016.77

**Published:** 2016-09-21

**Authors:** K Johannesson, R K Butlin

**Affiliations:** 1 Department of Marine Sciences, University of Gothenburg, Strömstad, Sweden; 2 Centre for Marine Evolutionary Biology, University of Gothenburg, Strömstad, Sweden; 3 Department of Animal and Plant Sciences, University of Sheffield, Sheffield, UK; 4 Stellenbosch Institute for Advanced Studies (STIAS), Wallenberg Research Centre at Stellenbosch University, Stellenbosch, South Africa

## Abstract

It is intriguing that conspicuous colour morphs of a prey species may be maintained at low frequencies alongside cryptic morphs. Negative frequency-dependent selection by predators using search images (‘apostatic selection') is often suggested without rejecting alternative explanations. Using a maximum likelihood approach we fitted predictions from models of genetic drift, migration, constant selection, heterozygote advantage or negative frequency-dependent selection to time-series data of colour frequencies in isolated populations of a marine snail (*Littorina saxatilis*), re-established with perturbed colour morph frequencies and followed for >20 generations. Snails of conspicuous colours (white, red, banded) are naturally rare in the study area (usually <10%) but frequencies were manipulated to levels of ~50% (one colour per population) in 8 populations at the start of the experiment in 1992. In 2013, frequencies had declined to ~15–45%. Drift alone could not explain these changes. Migration could not be rejected in any population, but required rates much higher than those recorded. Directional selection was rejected in three populations in favour of balancing selection. Heterozygote advantage and negative frequency-dependent selection could not be distinguished statistically, although overall the results favoured the latter. Populations varied idiosyncratically as mild or variable colour selection (3–11%) interacted with demographic stochasticity, and the overall conclusion was that multiple mechanisms may contribute to maintaining the polymorphisms.

## Introduction

In finite populations, genetic variation is reduced through the combined effects of drift and selection. Still, populations of many species are polymorphic for inherited traits that are likely to affect individual fitness, such as colour. Why species are polymorphic for colour and how this will influence their evolution are not at all clear (Wellenreuther *et al.*, 2014; Bolton *et al.*, 2015). Even in well-studied species and over short evolutionary timescales, it is often challenging to explain how colour polymorphisms are maintained (see, for example, Clarke, 1962; Cook, 1998, 2007; Cook and Saccheri, 2013; McLean and Stuart-Fox, 2014).

If colour does not affect fitness, or is under weak selection, a polymorphism will be more or less structured in time and space by gene flow, population demography and history. Indeed, many (perhaps the majority) of natural populations are likely to be far from equilibrium and also unstable polymorphisms may be sustained over extended periods of time owing to stochastic effects during colonization, range expansion and population perturbation (Cook, 1998; Excoffier *et al.*, 2009). Therefore, selection should only be invoked with caution when seeking to explain colour polymorphism.

Spatially variable selection in a heterogeneous environment in combination with gene flow may maintain a polymorphism following a simple population genetic model (Levene, 1953), and empirical support for this is extensive (reviewed in Hedrick, 1986, 2006). In contrast, polymorphisms maintained by selection within an essentially panmictic population living in a temporally and spatially homogeneous environment require a more complex mechanism. Two conceptually very attractive models that can explain a stable polymorphism within a homogeneous population are: (1) negative frequency-dependent selection and (2) heterozygote advantage.

In his classic review of the evolution of genetic diversity, Clarke (1979) argued that pervasive frequency-dependent selection was the best explanation for widespread polymorphism. Negative frequency-dependent selection implies that the relative fitness of a fixed phenotype will switch at some intermediate frequency between selection disfavouring and selection favouring the phenotype over other phenotypes (Clarke, 1979). Such frequency dependence can arise in many ways: clearcut cases are alleles for self-sterility in hermaphroditic plants that are selected against when common but not when rare (Castric and Vekemans, 2004) but Clarke (1979) offered many other potential underlying mechanisms. Negative frequency-dependent selection is also an attractive explanation because it can maintain multiple alleles, as demonstrated by very high numbers of alleles at the loci for self-incompatibility in plants (Delph and Kelly, 2014).

The view of Clarke (1979) on frequency dependence was formed partly through his early work on colour polymorphism in snails (Clarke, 1962, 1969). Visually hunting predators are thought to maintain colour polymorphism in prey species by negative frequency-dependent selection (‘apostatic selection', Clarke, 1962). Apostatic selection requires a mechanism in the predator that switches the predation strategy, and here search images and perceptual switching are critical components (Bond, 2007). There is convincing experimental support for negative frequency-dependent selection of colour polymorphic prey species in some specific cases (Endler, 1980; Reid, 1987; Jormalainen *et al.*, 1995; Olendorf *et al.*, 2006; Hughes *et al.*, 2013). There is also experimental evidence for visual predators having search images resulting in perceptual switching when a prey item becomes less common (Bond, 2007). However, whether or not frequency-dependent selection is a major mechanism maintaining colour polymorphisms in natural populations remains under debate, partly because of the lack of rigorous tests of alternative explanations (Punzalan *et al.*, 2005; Bond, 2007; Ibarra and Reader, 2013). In a few cases, combining empirical experiments and modelling of populations has generated strong support for apostatic selection maintaining a colour polymorphism, as in the example of sexual selection among female colour morphs in damselflies (Svensson *et al.*, 2005; Bots *et al.*, 2015; Le Rouzic *et al.*, 2015).

The alternative to negative frequency-dependent selection, heterozygote advantage, does not require a predator with perceptual switching, and is, in this respect, a more parsimonious model. However, heterozygote advantage generates a genetic load in the population by the production of suboptimal homozygotes, and it can only maintain multiple alleles in some special cases (Clarke, 1979). Hedrick (2012) reviewed support for heterozygote advantage and found only a few cases in natural populations. However, it is important to note that the advantage may not be solely due to the colour but could be influenced by other traits, perhaps affected by other loci in close association (see, for example, Gratten *et al.*, 2008).

Snails are classical objects for studies of colour polymorphisms (Goodhart, 1963; Clarke *et al.*, 1978) not least because colours tend to have a rather simple Mendelian inheritance (Cain and Sheppard, 1950; Cain, 1983; Palmer, 1985; Cook, 1998). Snail shell colour may be selectively neutral, affect metabolism during heating and/or cooling (Heath, 1975; Chang, 1991; Phifer-Rixey *et al.*, 2008) and provide camouflage with respect to visual predators (Reimchen, 1979; Reid, 1987; Johannesson and Ekendahl, 2002). Other factors have been found to correlate with colour, for example, salinity tolerance (Sokolova and Berger, 2000), shell strength (Rosin *et al.*, 2013), degree of parasite infection (Scheil *et al.*, 2014) and mate choice (Rolán-Alvarez and Ekendahl, 1996). It seems likely, however, that one or several of these correlations are explained by traits being linked to colour in a supergene or an inversion (Wellenreuther *et al.*, 2014).

**Figure 1:**
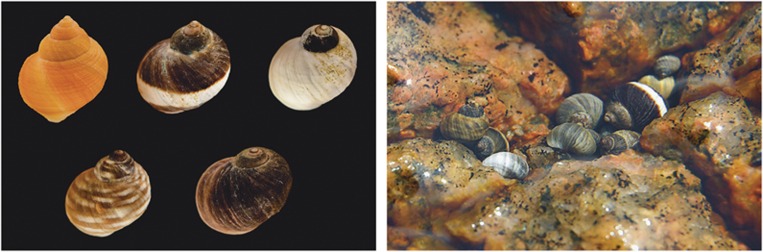
Left plate shows representative shell colours of Swedish *Littorina saxatilis* of the wave ecotype, with red, banded and white being more conspicuous whereas tessellated and black are considered more cryptic against the background. Right plate shows snails against a cliff background. Photo: Fredrik Pleijel and Patrik Larsson.

One particularly intriguing observation is the occurrence of rare and very conspicuous colour morphs in populations dominated by various cryptic colours (reviewed in Wellenreuther *et al.*, 2014). For example, in discussing apostatic selection, Clarke (1969) emphasized that not all colour morphs of *Cepaea* are cryptic. Populations of the marine snail *Littorina saxatilis* provide another clear case. Most populations are highly polymorphic for shell colour with cryptic colours ranging from dark to light brown being most common, but white, bright red and banded snails are found in low frequencies side by side with cryptic snails. In Swedish populations in wave-exposed cliff habitats, low frequencies (<10%) of white, red and banded snails are present, together with snails that are cryptic in different dark-brown colours (Ekendahl and Johannesson, 1997) (Figure 1). Notably, the distribution of the conspicuous colour morphs varies among geographic areas: in Sweden they are confined to wave-exposed cliff substrata, but these same morphs have higher frequencies and a wider distribution spanning several habitats, for example, in Iceland and NW Russia (Ekendahl and Johannesson, 1997).

We wanted to find out why the conspicuous colour morphs are maintained at low frequencies in *L. saxatilis* despite the fact that snails of these colours are likely to be less cryptic than other snails to visually hunting predators. Repeated sampling of a polymorphism over time, following natural or anthropogenic perturbation, is a powerful approach to estimate the strength of selection (see, for example, Endler, 1986; Kettlewell, 1961; Johannesson *et al.*, 1995), and can also be used to evaluate effects of drift and migration. We combined manipulation of several Swedish populations with time-series records to generate data that we compared with predictions from models of drift, migration, directional or balancing selection. A similar approach, but based on natural perturbations, was recently used by Le Rouzic *et al.* (2015) to estimate the strength of balancing selection maintaining female colour polymorphisms in populations of damselflies. Indeed, time-series data have a great advantage over estimates of selection from data on survival, fecundity and similar, in that they integrate lifetime fitness and reproduction and, in addition, account for minor fluctuations among generations.

We were not primarily interested in ‘Levene-type' polymorphisms and hence, to reduce the effects of migration (but still prevent excessive drift), we established panmictic populations (*N*=500–10 000) in small homogeneous and isolated habitats (small rocky skerries previously inhabited by snails, see below). To reduce effects of different demographic histories and to be able to assess the importance of contemporary stochastic factors, we started several parallel populations from the same well-mixed source population. To reveal the effects of selection (Endler, 1986), we perturbed frequencies of the conspicuous colour morphs from normal (1–6%) to high levels (~50%). Practically, experimental populations were started by transplantation of snails from a large island to small rocky skerries that had been cleared of snails by a toxic algal bloom a few years earlier. Open water acted as a barrier to dispersal among populations as this snail is confined to the intertidal, has no pelagic larvae and only occasionally disperses by rafting (Johannesson and Johannesson, 1995). Following the establishment of the experimental populations in 1992, we recorded the changes in colour frequencies over 21 years, including phenotyping more than 15 800 individuals over 11 sampling occasions. We used a maximum likelihood approach to compare the temporal changes in phenotype frequency with predictions from models of drift, migration, directional selection, heterozygote advantage or frequency-dependent selection.

## Materials and methods

### Study species

*Littorina saxatilis* is typically found in rocky habitats, but sometimes also in saltmarshes and brackish water lagoons (Reid, 1996). It is strikingly polymorphic in shell size, shape and colour, with shape/size morphs (‘ecotypes') strictly associated with different microhabitats (Johannesson *et al.*, 2010), whereas shell colour is usually polymorphic also within habitats (Reid, 1996). Juveniles and adults are limited by a lifetime displacement of a few metres only, and larval development is completed in a female brood pouch before juveniles are released on the shore. The present study includes only snails of the Swedish wave ecotype that live in densities of 100–1000 per m^2^ on cliff surfaces exposed to strong wave action. These snails have a maximum lifespan of 1–2 years after becoming sexually mature at 6–8 months (from observation in lab. cultures, K Johannesson). Females are receptive all year round and mate multiple males at an extreme rate, each female simultaneously carrying offspring sired by some 20 males (Panova *et al.*, 2010). Females may also use stored sperm for up to a year, and if one multiply-mated female is introduced to an empty site, this may be enough to establish a population of some thousand individuals a few generations later (Johannesson and Johannesson, 1995).

### The genetics of shell colour

Colour is one of the few phenotypic traits that can be conveniently assessed directly in the field, and in addition has a simple Mendelian mechanism of inheritance in many species, including species of gastropods (see, for example, Palmer, 1985; Richards *et al.*, 2013). In *L. saxatilis*, ground colour and banding are inherited by separate and apparently not closely linked loci with dominance for alleles coding for banded, white and red morphs (Atkinson and Warwick, 1983; Ekendahl and Johannesson, 1997; this study). Consequently, in our models banding was coded for by one locus with two alleles and dominance for band, and the different ground colours were also coded for by separate, unlinked loci, each with a dominant allele resulting in a conspicuous colour. We assumed independent phenotypic effects of the three loci except that the banded phenotype was not expressed in the presence of the white allele. (Occasionally, we found individuals, 11 in total, that had a red instead of a white band that may suggest that there is one additional locus coding for band colour with different alleles for white and red, but we ignored this information and considered banded as one morph.)

### Manipulations and sampling

**Figure 2:**
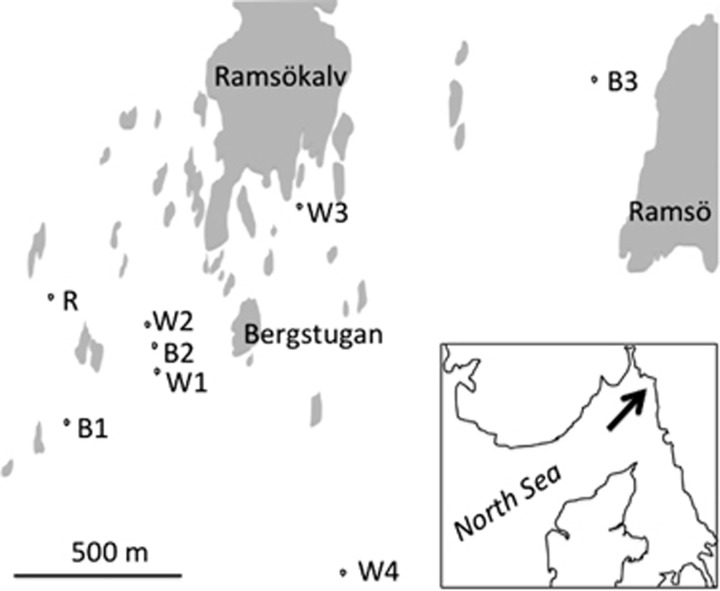
Map showing a small part of the archipelago on the Swedish west coast where the small intertidal skerries (2–10 m^2^ area) used in this study are situated. The experimental snails were all from the island Bergstugan. Replicate skerries were manipulated for white (W1–W4) and banded (B1–B3), and one skerry was manipulated for red (R).

In 1988, an extreme bloom of a toxic marine phytoplankton (*Chrysochromulina polylepis*) completely wiped out populations of *L. saxatilis* inhabiting small and shallow rocky skerries 2–10 m^2^ in area that were earlier inhabited by populations of several hundred to several thousand snails (Johannesson and Johannesson, 1995). Populations inhabiting larger islands were also heavily depleted, but snails high up in the intertidal survived as they were above the toxic water. In 1992, populations on islands had mostly recovered whereas the majority of shallow skerries remained empty because of low rates of spontaneous recolonization. In that year, we transplanted snails of rare and conspicuous colour morphs (white, red and banded) from an island (Bergstugan, N 58° 49' 19", E 11° 2' 19" on the Swedish west coast), where these morphs occurred in low frequencies, to nine nearby skerries (Figure 2, and see Supplementary Table S1 for details). Snails of the same colour were thoroughly mixed and 135 white adult snails were released on each of five skerries, 126 banded adult snails were released on each of three skerries and 65 red adult snails were released on a single skerry. All nine transplants were successful in that 4 months later populations were established and numbers had increased to 150–500 snails. One of the populations manipulated for white, however, died out after some years and was not included in the analyses presented here. Another skerry (White-4, also manipulated for white), experienced a severe bottleneck a few years after establishment, but recovered and reached a high population size that persisted for the remainder of the observation period. We used 1996, after the bottleneck, as the first sample for this site.

Following the transplantation (1 June 1992), we revisited all skerries on 11 successive occasions until 2013, and scored the colour of 200 adult snails (~2–5 mm) per population (or as many as found for *N*<200) from two separate areas of each skerry. Average sample sizes were 163, 198 and 168 for white, red and banded populations, respectively, over the 11 sampling occasions. Snails were phenotyped in the field for colour (white, red, banded and cryptic), and carefully returned to the skerry in a way that allowed them to attach again. The same person (KJ) did all the sampling and the scoring of colours, although in most years assisted by a second person. Total population size was estimated at each sampling occasion by approximate counting of all adult snails inhabiting the skerry.

### Analysis of populations and pools of populations

We used explicit population genetic modelling based on the assumption that the conspicuous colours (white and red) and banding were genetically coded as outlined above. Where we had several populations manipulated for the same colour, model fits were also conducted over all populations with parameters the same for all sites (‘pooled-I'), and compared with the ‘combined' fits of individual populations. This provided a test for equality of fitted parameters across populations. In addition, we pooled populations but allowed for site-specific initial frequencies, whereas other parameters were common across pooled populations (‘pooled-II').

### Starting allele frequencies

We included the allele frequency at the time of the first sample as a parameter (*p*_0_) to estimate in each fitted model, along with other parameters. For the introduced individuals, we expected the frequency of the colour allele to be 0.5+*p*/2 (*p* being the frequency of the dominant ‘colour-allele' in the source population) as all snails had at least one colour allele, and the other allele was also a colour allele with frequency *p*. Because of multiple mating and long-term sperm storage, the introduced females also carried sperm and embryos from matings with males of the donor population that had a frequency of the colour alleles that was *p*. Upon arrival to the skerry, however, new matings with males that had colour frequency 0.5+*p*/2 increasingly contributed to the genotypes of the offspring. Hence, if all offspring in the first sample derived from matings before the transplant, we expected the allele frequencies of the first offspring generation to be *p*_0_=((0.5+*p*/2)+*p*)/2=0.25+0.75*p* but if all were instead derived from introduced males we would expect *p*_0_=0.5+*p*/2 (with coloured phenotypes at a frequency of *P*=*p*_0_^2^+2*p*_0_(1−*p*_0_)). As we did not know the mix of pre- and post-introduction fathers, we instead fitted *p*_0_. Although genotypes in the introduced snails were clearly not in Hardy–Weinberg equilibrium, we assumed random mating with respect to colour and genotype frequencies close to equilibrium in subsequent samples.

### Model tests

The change in frequency over time for each of the three conspicuous colour morphs was fitted to predictions from different models. In all the models we assumed populations to be in Hardy–Weinberg equilibrium before selection. We used a generation time of 1 year and non-overlapping generations. This was a simplification although close to reality as only a small proportion of the snails seem to survive ⩾2 years. The error distribution for the observed frequencies was assumed to be binomial, reflecting even distribution on each skerry and random sampling. As the observed data were in the form of counts of the focal colour morph and of all other snails, this makes appropriate allowance for variation in sample size.

First, we tested the null hypothesis that genetic drift alone could account for the temporal changes in colour phenotype frequencies that we observed. With drift (and sampling error) as the only factors influencing changes in phenotype frequency, we predicted that a regression of *P*' on *P* would have slope 1 and zero intercept (*P* here being the phenotype frequency in one sample and *P*' the frequency in the next sample), as the expected value of *P*' is *P*, whatever the value of *P*. Variation around this expectation depends on the effective population size and the sampling error. Because we could not separate these components, we did not try to estimate the effect of drift but only to test whether drift alone was sufficient to explain our observations. Each data point (*P*, *P*') is independent under the null hypothesis because change in frequency in one time interval does not influence change in the next interval. We then fitted the regression for phenotype frequencies using a logit scale with binomial errors (using the glm function in R). On this scale, the expectation is also a slope of 1 and intercept of zero (equivalent to *P*=0.5, *P*'=0.5). R version 3.2.2 was used in all analyses (R Core Team, 2014).

As the model of drift alone was generally rejected (see below), we proceeded with fitting additional models, one of migration and three of selection, to our empirical data. Note that these models do not exclude drift that contributes to variation around the expectation. We fitted models using the mle2 function in the bbmle package in R (optimizer: optim, method=‘L-BFGS-B' to allow limits on parameter ranges, where needed, otherwise using the default method) (https://cran.r-project.org/web/packages/bbmle/index.html). We did not attempt to fit migration and selection simultaneously because there is unlikely to be sufficient information in the data to distinguish their effects.

In the migration model (M), we calculated the proportion of each generation made up of immigrants (*m*) required to explain the decline in frequencies of rare colour morphs that we observed. Given *p*_t+1_=*mp*_eq_+(1−*m*)*p*_t_ (*p*_t_ is the frequency in generation *t*, iterated for the number of generations between samples to provide expected frequencies), we needed to estimate only *m* as the equilibrium allele frequency (*p*_eq_) in the source population of migrants could be obtained from the other skerries. Nonfocal colour alleles (see, for example, banded alleles in a white skerry population) were introduced to skerries at frequencies close to equilibrium populations and had presumably remained near equilibrium frequencies (assuming the alleles responsible for the different colour morphs were at different loci and the polymorphism was maintained independently by selection). Hence, we used the mean frequency of a morph in all nonfocal populations to estimate equilibrium frequencies and searched for the maximum likelihood value of *m* for each focal skerry, given the observed phenotype frequencies.

In the model of directional selection (DS), we assumed full dominance of colour alleles (*w*_AA_=*w*_Aa_=*w*_colour_), whereas the fitness of the recessive homozygote, all other colours, was fixed (*w*_aa_=1). We found the maximum likelihood estimate of *w*_colour_ using the iteration: *p*_t+1_=[*w*_colour_*p*_t_^2^+*w*_colour_*p*_t_(1−*p*_t_)]/[*w*_colour_*p*_t_^2^+*w*_colour_*p*_t_(1−*p*_t_)+ (1−*p*_t_)^2^]. The strength of selection against the conspicuous colour, *s*, was obtained from the fitness estimate (*s=*1−*w*_colour_).

In the model with potential for heterozygote advantage (HA) the fitnesses of the underlying homozygous genotypes at the colour locus (*w*_AA_ and *w*_aa_) were fitted in relation to the heterozygote fitness (*w*_Aa_=1) using the iteration: *p*_t+1_=[*w*_AA_*p*_t_^2^+*p*_t_(1−*p*_t_)] / [*w*_AA_*p*_t_^2^+*p*_t_(1−*p*_t_)+*w*_aa_(1−*p*_t_)^2^]. Note that heterozygote advantage, and hence balanced polymorphism, is only present where *w*_AA_<1 and *w*_aa_<1. Where these conditions were satisfied, equilibrium allele frequencies (*p*_eq_*=w*_aa_ / [*w*_AA_+*w*_aa_]) were calculated and used to predict the frequency of the conspicuous colour morph at equilibrium.

Finally, in the model assuming frequency-dependent selection (FD) we set the fitness of the other colour morphs (recessive homozygotes) to unity (*w*_aa_=1) and allowed the fitness of the conspicuous colour morph (homozygotes and heterozygotes) to vary with phenotype frequency: *w*_colour_=*w*_AA_=*w*_Aa_=*a*+*bP*, where *P* is the phenotype frequency of the conspicuous morph (*P*=(*p*^2^+2*p*[1−*p*])). Allele frequencies were then iterated as for the DS model. At equilibrium *P*=(1−*a*)/*b*, making the fitnesses of the two phenotypes equal, and *a*>1 and *b*<0 is required for the polymorphism to be stable.

In all cases, approximate confidence intervals were obtained by the profile likelihood method, using the *confint* command in R. Models were compared using χ^2^=−2ΔLL, with degrees of freedom equal to the difference in the number of parameters, for nested models, and the Akaike information criterion otherwise, obtained using the *AIC* command in R. Where multiple tests of the same hypothesis were made (for example, over the four skerries manipulated for the white morph), we adjusted the critical probability using the sequential Bonferroni method.

## Results

**Figure 3:**
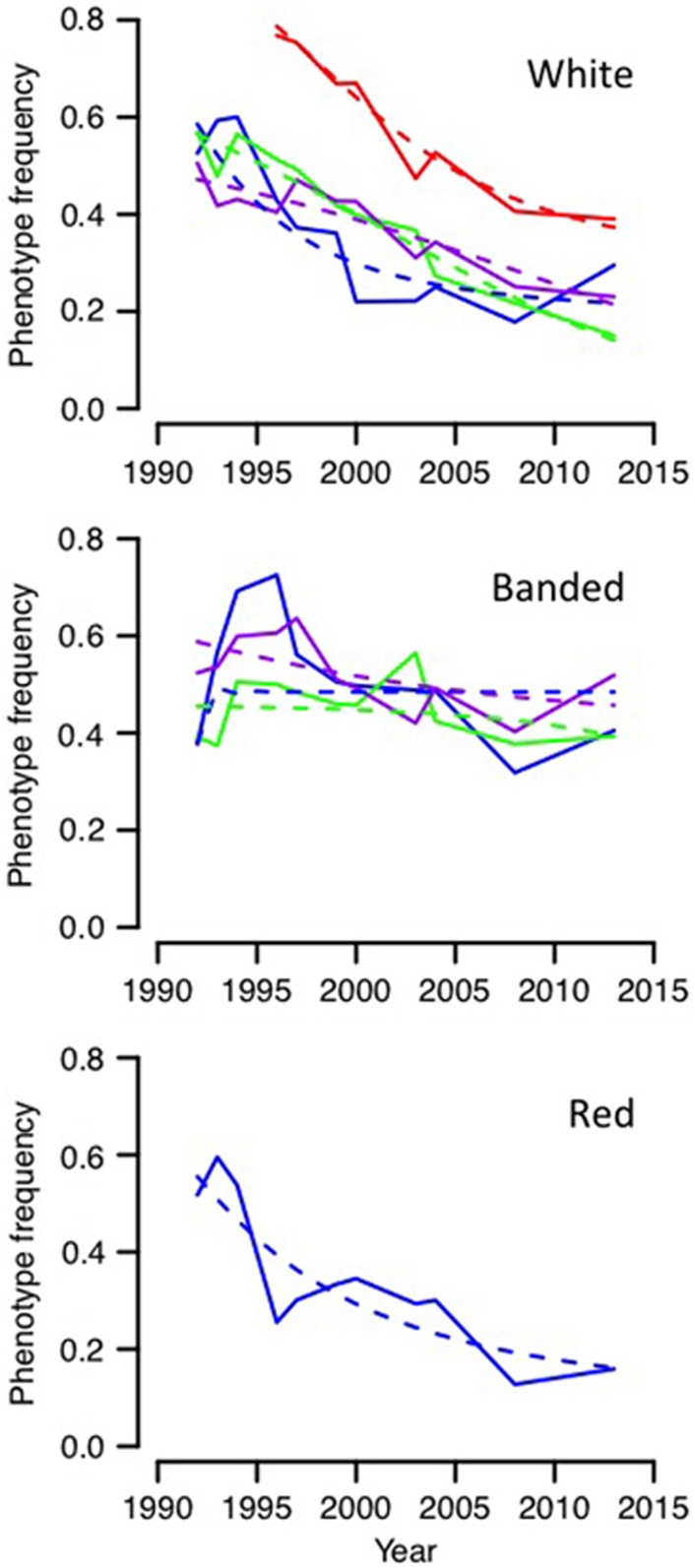
Frequencies of colour phenotypes in skerry populations from 4 months after the manipulation in 1992 to 2013 (solid lines). Fitted declines in frequencies under a model of frequency-dependent selection (broken lines). Populations are coded as follows. (Top) White-1, blue; White-2, green; White-3, purple; White-4, red. (Mid) Banded-1, blue; Banded-2, green; Banded-3, purple. (Bottom) Red, blue.

Overall, we observed that the frequencies of the different conspicuous colour morphs declined over the time period of observations (21 years). The decline was most obvious in populations manipulated for red and white colours, and less obvious in the banded populations (Figure 3). Colour frequencies fluctuated, sometimes quite substantially and in some cases linked to population bottlenecks (see below).

### Starting allele and phenotype frequencies

With the exception of one population (White-4) that early on passed through a strong bottleneck that shifted colour allele frequencies upwards (see below), the fitted starting allele frequencies of the colour alleles (*p*_0_) were all between 0.26 and 0.36, resulting in phenotype frequencies from 0.38 to 0.59 (with little variation is estimates among models; Supplementary Table S2). This indicated that the first generations of snails born on the skerries were mainly a result of pre-introduction matings. After the introduction, females started to mate with males of the manipulated population that had a much higher frequency of the colour allele than males of the source populations. Therefore, we expected that both phenotype and allele frequencies would increase somewhat during the first few generations. Indeed, a slight increase in colour morph frequencies following establishment was observed in several of the populations (Figure 3) but, for simplicity, we did not include this in our models.

### Variation within populations

We found significant heterogeneity in colour frequencies between the two samples taken within each skerry in only 2 out of 69 pairwise comparisons (*χ*^2^ tests corrected for multiple testing, Supplementary Table S3). This suggested that the colour frequencies were largely homogeneous over the whole of each skerry.

### Variation among population*s*

Among the populations manipulated for white colour, the sum of the log likelihood estimates for individual skerries (‘white combined') gave a much better fit (lower −2LL value) than the pooled data in which we used the same initial frequency of the colour allele (‘pooled-I') (Supplementary Table S4). This was true for all four models (*χ*^2^ for M=237, DS=229, d.f.=6; HA=262, FD=265; d.f.=9; *P*<<0.001 in all cases) and implies that there were idiosyncratic patterns of phenotype variation for each population. Using site-specific initial allele frequencies (‘pooled-II') increased the fit of the models somewhat compared with a shared starting frequency (pooled-I), but the combined estimates still gave much better fits (*χ*^2^ for M=63, DS=36, d.f.=3; HA=71, FD=76, d.f.=6; *P*<<0.001 in all cases). In conclusion, the four populations manipulated for white colour behaved differently both with respect to the pattern of frequency changes and the starting frequencies of the white morph.

In the three populations manipulated for banded colour, sums of the individual population models (banded combined) again fitted the data much better than when pooling data using the same starting allele frequency (pooled-I) (*χ*^2^ for M=34, DS=21, d.f.=4; HA=35, FD=34, d.f.=6; *P*<<0.001 in all cases). The combined model gave better fits than the model using pooled data and site-specific starting allele frequencies in the cases of directional selection, heterozygote advantage and frequency dependence, although not significantly so in the migration model (*χ*^2^ for M=5.65, d.f.=2, NS; DS=6.67, d.f.=2, *P*<0.05; FD=10.08, d.f.=4, *P*<0.05; HA=6.70, d.f.=4, *P*<0.05). This suggests that, also for the banded populations, the initial frequencies varied among populations but patterns of frequency change of the banded morph tended to be more similar among populations than was the case for the white morph as the improvements relative to the pooled-II model were less extreme.

### Effects of drift

All finite populations are affected by drift. Therefore, we specifically tested whether the changes in phenotype frequencies that we observed were more consistently directional than could be explained by drift alone. We found that in all but two populations (among white, banded and red), an unconstrained regression of *P*' on *P* was a better fit to the data than the drift expectation (Table 1). In the White-1 and White-3 populations, fitted regressions were similar to most other sites, although not significantly different from expectation. Thus, we concluded that there was evidence to reject drift alone as an explanation for the observed declines of conspicuous morph frequencies.

Under balancing selection, one would expect slopes less than unity because this means that it is possible, at some value of *P*, for the change in frequency to be zero and the equilibrium to be stable. In three of the four white populations, and in the red population, the fitted slope was <1.0 and an equilibrium colour frequency around 0.25 was predicted (Table 1). Similarly, in two of the banded populations the slopes suggested balancing selection but in these cases equilibrium frequencies around 0.50 were suggested (Table 1).

We also applied this test to the unmanipulated conspicuous colour morphs on each experimental skerry. There was a significant departure from the drift expectation in 8/15 tests, 4 of the nonsignificant cases being for the red morph where low frequencies make estimation unreliable (Supplementary Table S5). In four of the cases where drift was rejected, the slope was below 1, indicating balancing selection. Thus, the temporal variation in frequencies of conspicuous colour morphs in unmanipulated populations also suggested that these colours were under selection, although evidence for balancing selection was equivocal.

Although drift alone could not explain the observed changes in phenotype frequencies, population bottlenecks potentially caused large shifts in allele frequencies. We noted five bottlenecks in total with estimated population sizes <100 individuals (Supplementary Figure S1). During three of these bottlenecks, frequencies of the focal colour morphs increased. Thus, for example, population White-4 passed through a bottleneck in 1993 and 1994, with 30 and 0 snails found at sampling, respectively, and this was associated with a 20% increase in frequency of the white morph from 1992 to 1996 (note that model fits for this skerry used data from 1996 onwards). Similarly, the banded morph increased by 35% in population Banded-3 during a bottleneck (68 individuals found 1994).

### Migration

As frequencies of conspicuous colours were much higher in the manipulated skerry populations than in islands surrounding the skerries, rafting of snails from nearby islands would decrease frequencies of white, banded and red on the skerries over time. Using background frequencies of colour morphs, we fitted a model of migration to the empirical data. We estimated equilibrium frequencies of each colour allele from mean frequencies of colour morphs in all nonfocal populations (0.024, 0.013 and 0.005 for white, banded and red alleles, respectively, corresponding to phenotype frequencies of 4.8, 2.7, and 1%).

In none of the populations did the migration model give a significantly better fit to the empirical data than all other models. However, in two white and the red populations the migration model was preferred over the directional selection model (although at a lower likelihood than HA and FD models) (Table 2 and see below). Furthermore, estimated rates of immigration (*m*) that would explain the observed decay in colour frequencies in the skerry populations were generally high, that is, 4–7% in white and red populations and 0.5–2% in banded populations (Table 3). From the average population size of all skerries (*N*=1500), this translates to 60–105 migrants received per population per generation (*Nm*), on average, in the white and red, and 7.5–30 in the banded populations.

### Directional selection

The model of directional selection showed generally poorer fits than other models, with the exception that it gave a better fit to the data than the model of migration in one population (White-2) (Table 2). Indeed, in three populations, directional selection could be rejected in favour of either one or the other of the two models that permit balancing selection (White-1, White-4 and Red) (Table 2). However, assuming directional selection, we were able to estimate selection coefficients (*s*) against the conspicuous phenotypes: in the white and red phenotypes these were in the range 6–11%, whereas in the banded phenotype they were 3–4% (Table 3).

### Heterozygote advantage

The model with potential heterozygote advantage gave better fits to the data than did the migration model in two white populations and (marginally) in the red population, and it gave better fits than the directional selection model in three populations (two white and the red) (Table 2). Protected polymorphism was predicted for White-1, White-4 and Banded-3 and was compatible with the fitted parameters (confidence intervals for both fitness parameters included values <1) also for White-3, Banded-1 and Red (Table 3). For Banded-2 the fits were uninformative.

### Frequency-dependent selection

The model of frequency-dependent selection showed a very similar result to the model of heterozygote advantage and revealed significantly better fits than the models of migration in White-1, White-4 and marginally in Red populations, as well as in the white combined analysis (Table 2). This model was also a better model than directional selection in populations White-1, White-2 and Red, and in white combined (Table 2). Parameter values were consistent with protected polymorphism (*a*>1, *b*<0) in the same skerries as for the HA model, with clearer evidence in this case for Red (Table 3). Thus, overall, there was strong support for balancing selection in two of the white populations and in the red population, whereas in the two remaining white populations one was equivocal and FD was suggested in the other but not a polymorphic equilibrium. Among the banded populations, fits were very similar among models. To discriminate between the models of heterozygote advantage and frequency-dependent selection was difficult, but in terms of absolute values the model of frequency-dependence gave the best fits in all except the Red population (Table 2).

### Equilibrium frequencies of conspicuous morphs

From the populations with model support for balancing selection, we derived predictions of equilibrium frequencies of colour alleles and phenotypes. These estimates diverged substantially among populations manipulated for different colour morphs, with white phenotypes reaching a plateau at 17–30% where polymorphism was predicted, banded already at 39–48%, whereas red were predicted to reach equilibrium at 7%. Although the observed frequencies of the colour morphs in nonfocal skerries were very variable, they were much lower on average (white 5%, banded 3% and red 1%, Supplementary Table S6) than these model predictions. However, these observed values were very close to the overall average frequencies of the colour morphs in the area (white 6%, banded 3% and red 1%, Supplementary Table S7).

Noting the large discrepancy between background values and those observed from the model, we tested whether a model of frequency-dependent selection eventually reaching the background frequencies would give a better fit to the data than an unconstrained model. We used the observed frequency of white on nonfocal skerries (0.0482) and constrained the model using *a*>1 and *b*=(1−*a*)/0.0482. However, the constrained model did worse than the model in which the equilibrium frequency was unconstrained in all comparisons, significantly so for two of the four white populations and for the summarized data (Supplementary Table S8). Using a similar approach for the red population, the constrained fit (−2LL=109.03) was very similar to the unconstrained fit (108.6, Table 2), suggesting evolution towards the expected equilibrium in this population. We did not evaluate this possibility for the banded populations as the overall support for balancing selection was less strong in this case.

## Discussion

The manipulative increase of rare and conspicuous colour morphs of *L. saxatilis* to extreme frequencies was followed by a decline in frequency over the following 21 years in all populations, suggesting a prime role of selection on these colours. However, drift seems to be important in shaping colour frequencies in *Cepaea* land snails (reviewed in Cook, 1998; Bellido *et al.*, 2002). In particular, during establishment and range expansion, drift can be a strong evolutionary force that occasionally also promotes otherwise rare alleles and phenotypes (Excoffier *et al.*, 2009). Establishment of local populations of *L. saxatilis* in an archipelago of hundreds of small islands (as in the study area) will include repeated bottlenecks but their primary effect will be to remove rare alleles (Barton and Charlesworth, 1984). Thus, rare colours are more likely here than elsewhere to be lost by drift, unless protected by selection. However, high levels of multiple mating and long-term sperm storage in females of *L. saxatilis* buffer against stochastic loss during bottlenecks (Rafajlovic *et al.*, 2013).

Despite the fact that all populations manipulated for the same colour came from the same well-mixed pool of snails, allele and phenotype frequencies already differed among populations in the first few generations. There may be several stochastic reasons behind this effect, including sampling effects for proportions of homozygotes for the colour allele among transplanted individuals, different proportions of pre- and post-transplanted matings and early demographic fluctuations in population sizes. In addition, minor habitat differences may have led to some differential selection among skerries.

Although migration was rejected in 2–3 of the populations, we could not exclude it as the cause of frequency decline in several of the populations. However, the levels of migration needed to explain the empirical data were high for a sedentary species without larval dispersal, with 60–105 immigrants per generation in white and red populations and 7.5–30 in banded populations. Observations of migration made from rates of recolonization of empty skerries during the same period of time concluded that migration to a skerry was in the range of 0.05–0.1 immigrants per generation (from data in Johannesson and Johannesson, 1995). The migration rate estimated from genetic differentiation among island populations of the area using genome scans of thousands of markers was ~2 immigrants per generation (Ravinet *et al.*, 2016). Therefore, overall, migration is likely to have contributed to the gradual decline in colour frequencies but only to a minor degree. It could also contribute to the maintenance of polymorphism if there were directional selection against conspicuous colour morphs on the skerries, but not elsewhere in the archipelago. Therefore, it would be desirable to model selection and migration together but we doubt whether the observations are sufficient to discriminate their effects.

Our results indicate contemporary selection against high frequencies of conspicuous colour morphs, and this corroborates earlier experimental studies showing selection favouring crypsis in littorinid snails (Reimchen, 1979; Reid, 1987; Johannesson and Ekendahl, 2002). Indirect support for selection on shell colour in *L. saxatilis* also comes from the correlation of colour frequencies with wave exposure, type of substratum and sometimes shore level (Ekendahl and Johannesson, 1997). Although the distribution of colour morphs may also be shaped by selection for thermal tolerance, as shown for light and dark colours of *Littorina obtusata* (Phifer-Rixey *et al.*, 2008), the thermal regimes of our study were similar within and among skerries and trends of loss of conspicuous colours were similar in both white and dark (banded and red) snails, and hence selection for thermal tolerance seems unlikely in this case.

The estimated strength of selection varied among colour morphs with the banded morph having 3–4% lower fitness than the cryptic morph, whereas white had 6–11% and red 10% lower fitness (relative fitness in DS model, Table 3). These coefficients of selection are mild in comparison with strengths of selection on conspicuous Icelandic colours morphs of *L. saxatilis* on a background of seaweeds (40–75%, from data in Johannesson and Ekendahl (2002), or compared with selection on colour morphs in *Cepaea nemoralis* (20–50%) when introduced to new habitats (Ozgo and Kinnison, 2008; Ozgo, 2011).

We found both models of potentially balancing selection (heterozygote advantage and negative frequency dependence) to give an overall better fit to the data than did the model of fixed directional selection on colour. This was particularly true for the white and red phenotypes but the fits did not predict polymorphic equilibrium in all localities for white. The two models cannot be separated statistically but we note that the frequency-dependent model requires only the observed phenotypic variation in colour, whereas the model of heterozygote advantage requires some unobserved difference between heterozygotes and homozygotes at the colour locus or a nearby locus. In this sense, the FD model is more parsimonious. The presence of conspicuous colour morphs in practically all wave-exposed cliff habitats of the study area (Supplementary Table S6) indirectly supports balancing over directional selection. However, the conspicuous colour morphs (with the exception of red) are completely absent in boulder habitats (Ekendahl and Johannesson, 1997; extensive unpublished data), and hence it appears evident that in these habitats there is directional selection against white and banded colours even though alleles of the conspicuous colours are repeatedly introduced by gene flow between snails of different habitats (Panova *et al.*, 2006, and see Supplementary Figure S2).

Crabs are the main predators of *L. saxatilis* in Swedish boulder shores (Johannesson, 1986) but they seem to ignore the snail colour during hunting (Ekendahl, 1998). On wave-exposed cliff surfaces, birds (and possibly fish) are the main predators and it seems likely that these visually hunting predators were the main agents of colour selection on the manipulated populations.

Thus, balancing selection is both supported by the model tests and by the more general observation of a strong correlation between colour morph frequencies and habitat type in the study area. However, where polymorphism was predicted, the model predictions of equilibrium phenotype frequencies of the colour morphs were surprisingly high: 17–30% for white, 39–48% for banded and 7% for the red morph, whereas equilibrium frequencies of island and skerry populations were ~6% for white, ~3% for banded and ~1% for red (although variable, see Supplementary Table S7). The differences are clearly too large to be due to chance choice of skerries with atypical environments. It seems likely that the ‘true' equilibrium frequencies for white and banded, in particular, are much lower than those predicted from the models of balancing selection, and that the conspicuous colours have not yet reached equilibrium. The fits may tend to return estimates that predict equilibria around the current values, partly because the initial decline will be rather similar for models with different final frequencies: that is, only small changes in parameters are needed to alter the equilibrium and these small changes may not alter the overall fit by much.

One additional possibility is that the habitats of the skerries are generally different from, or more variable than, those of the larger islands. For example, the skerries are all very shallow and covered completely during high tide or strong wave action, whereas many snails on larger islands live in the splash zone above high tide. However, if the skerry habitats were more favourable to white and banded colour morphs than the islands, we would have noticed an increase in these morphs in the non-target skerry populations but these frequencies remained fluctuating at low levels (1–7%, Supplementary Table S6).

Negative frequency-dependent selection, because of apostatic selection by predators, is often suggested as the prime alternative for maintenance of colour polymorphisms in prey species under visual selection without formal rejection of alternative models (see reviews by Cook, 1998; Ibarra and Reader, 2013). Having permanently low frequencies in combination with being conspicuous to visual predators, the red, white and banded morphs of *L. saxatilis* may seem to be an obvious case of apostatic selection. However, despite using replicate time-series data including >15 800 observations, following strong perturbation events in populations with mild impacts of drift and migration the results were still partly equivocal. Although migration and drift could be excluded as sole drivers, this was primarily because we could combine model results with observations of migration and population demography. A large challenge was to discriminate between different types of selection. Time-series analysis following perturbation of genotype frequencies, or of the environment, is a powerful approach to detect selection and estimate its strength (Endler, 1986; Le Rouzic *et al.*, 2015) but it is limited in its ability to discriminate among alternative modes of selection or to incorporate migration and selection in the same model. Our approach has allowed us to conclude in favour of some form of balancing selection in most populations but support for this model was not strong and we were unable to discriminate between heterozygote advantage and negative frequency-dependent selection. Although we noted a weak tendency towards a generally better fit of the frequency-dependent model, being in absolute terms the best fit in 7 of 8 comparisons (bold figures in Table 2), the model predictions are simply too similar and hence these forms of selection must be separated using direct measures of fitness components under different conditions. Variation in the proximate mechanisms of selection among local populations, or different mechanisms of selection acting simultaneously within populations, as suggested in other systems (Oxford, 2005; Hedrick, 2012; Smith *et al.*, 2012), only makes the problem more difficult. Thus we cannot exclude the possibility that there are other (presumably more complex) models that more accurately describe this colour polymorphism. Either way, we have shown here that time-series data combined with explicit genetic modelling is a useful approach to increase our understanding of polymorphisms in wild populations.

## Data archiving

Colour phenotype numbers and sample sizes for all skerries over all years are available from the Dryad Digital Repository: http://dx.doi.org/10.5061/dryad.427p0.

## Figures and Tables

**Table 1:** Tests for changes in colour frequencies after manipulation (see Figure 3) that are not consistent with drift (that is, significance indicates effects larger than drift alone)

*Colour*	*Intercept*	*Slope*	n	*χ*^*2*^ *(d.f.=2)*	*Inferred equilibrium phenotype frequency*
White-1	−0.180±0.086	0.812±0.098	9	4.64	0.277
White-2	−0.157±0.054	1.135±0.106	9	17.16***	—
White-3	−0.175±0.078	0.843±0.153	9	6.56^†^	0.247
White-4	−0.151±0.080	0.879±0.120	7	8.46*	0.223
Banded-1	−0.037±0.055	0.414±0.123	9	21.97***	0.484
Banded-2	−0.172±0.059	0.331±0.201	9	12.66**	0.436
Banded-3	0.061±0.048	0.452±0.155	9	12.55**	0.528
Red	−0.324±0.068	0.707±0.082	9	23.23***	0.249

Intercept and slope from a regression of phenotype frequency in a sample (*P*') on frequency in the preceding sample (*P*) (estimates on logit scale). Where the slope is <1 (that is, drift alone is rejected), an equilibrium can be inferred where the fitted regression crosses the line of no change (*P*'=*P*).

^†^*P*<0.1, *P<0.05, **P<0.01, ***P<0.001, χ^2^ test for improvement of the fitted model over the drift expectation of zero intercept and slope of 1 (after sequential Bonferroni correction).

**Table 2:** Fits of models to empirical data on changing colour frequencies (Figure 3) and comparisons between models

*Colour*	*Model log likelihood (*−*2LL) estimates*				*Model comparisons*					
	*Migration*	*Directional selection*	*Heterozyg. advantage*	*Frequency dependent*	***M** vs **DS***	***M** vs **HA***	***M** vs **FD***	***DS** vs **HA***	***DS** vs **FD***	***HA** vs **FD***
Parameters/test	2	2	3	3	ΔAIC	χ_1_^2^	χ_1_^2^	χ_1_^2^	χ_1_^2^	ΔAIC
White-1	104.3	117.7	87.8	**85.6**	−13.4	16.4***	18.6***	29.8***	32.0***	2.2
White-2	75.6	70.0	70.0	**69.6**	5.5	5.5†	6.0*	0.0	0.5	0.5
White-3	73.7	72.2	72.2	**71.4**	1.5	1.5	2.3	0.0	0.9	0.9
White-4	53.9	58.5	52.4	**51.7**	−4.7	1.5	2.2	6.2*	6.8**	0.6
White combined	307.3	318.4	282.4	278.3	−11.1	24.9***	29.1***	36.0***	40.1***	4.1
Banded-1	99.5	98.8	98.8	**98.4**	0.7	0.7	1.1	0.0	0.4	0.4
Banded-2	80.7	80.7	80.7	**78.9**	0.1	0.1	1.9	0.0	1.8	1.8
Banded-3	86.9	87.3	86.3	**86.1**	−0.4	0.6	0.8	1.0	1.2	0.2
Banded combined	267.2	266.8	265.7	263.3	0.4	1.4	3.9	1.0	3.4	2.4
Red	111.6	116.8	**108.0**	108.6	−5.2	3.6†	3.0†	8.9**	8.2**	−0.6

Abbreviations: AIC, Akaike information criterion; DS, directional selection; FD, frequency-dependent selection; HA, heterozygote advantage; Heterozyg., heterozygote; M, migration.

Negative values indicate that the latter of the two models in the comparison had the higher likelihood value. In χ^2^ tests; d.f.=1 for single population model comparisons, d.f.=4 for White combined and d.f.=3 for banded combined. Bold log-likelihood estimates indicate the best models.

^†^*P*<0.1, *P<0.05, **P<0.01, ***P<0.001 (after sequential Bonferroni correction for repeated tests of white and banded skerries).

**Table 3:** Inferred parameter estimates and their confidence intervals (CIs) for the different models

	*Migration*	*Directional selection*	*Heterozygote advantage*		*Frequency dependent*	
*Population*	*Rate of migration,* m	*Relative fitness colour phenotype,* w_*AA,Aa*_	*Relative fitness colour homozygote (*w_*AA*_)^a^	*Relative fitness cryptic homozygote (*w_*aa*_)^b^	*Parameter* a *in* w_*AA,Aa*_*=*a+bP	*Parameter* b *in* w_*AA,Aa*_*=*a+bP
White-1	0.072 (0.057–0.086)	0.92 (0.90–0.94)	0.30 (0.05–0.54)	0.93 (0.86–0.98)	1.14 (1.06–1.24)	−0.70 (−0.99–(−0.45))
White-2	0.071 (0.060–0.084)	0.90 (0.88–0.91)	1.00 (0.84–NA)	1.12 (1.06–1.14)	0.87 (0.78–0.95)	0.075 (−0.14–0.30)
White-3	0.042 (0.030–0.054)	0.94 (0.93–0.96)	1.00 (0.76–NA)	1.06 (0.99–1.08)	0.88 (0.77–1.02)	0.17 (−0.20–0.57)
White-4	0.061 (0.047–0.074)	0.89 (0.86–0.91)	0.69 (0.50–0.92)	0.97 (0.87–1.09)	1.16 (0.96–1.36)	−0.52 (−0.89–(–0.14))
Banded-1	0.023 (0.013–0.033)	0.96 (0.94–0.98)	1.00 (0.78–NA)	1.04 (0.95–1.07)	*4.36*	*–6.93*
Banded-2	0.005 (0.000–0.016)	0.97 (0.97–1.01)	1.00 (NA–NA)	1.01 (NA–NA)	*0.69*	*0.67*
Banded-3	0.015 (0.007–0.024)	0.97 (0.96–0.99)	0.81 (0.46–NA)	0.95 (0.78–1.04)	1.13 (0.83–1.47)	−0.31 (−0.95–0.29)
Red	0.070 (0.061–0.086)	0.90 (0.88–0.92)	0.53 (NA–0.82)	1.00 (0.94–1.07)	1.03 (0.94–1.13)	−0.42 (−0.72–(−0.13))

Unreliable estimates due to poor convergence of the model fit are in italics and without CI. NA means that values reached predefined model bounds or could not be estimated. For starting frequency estimates see Supplementary Table S2.

a Lower bound 0.001, upper bound 1.000.

b Lower bound 0.001.
