# Supplementary material for: What explains rare and conspicuous colours in a snail? A test of time-series data against models of drift, migration or selection
Source: Heredity (Edinb). 2016 Sep 21;118(1):21–30. doi: 10.1038/hdy.2016.77 (PMC5176118; doi:10.1038/hdy.2016.77)

**Supplementary information**

Table S1. Exact positions of skerries, approximate area and average estimated population sizes over sampling occasions.

| Skerry | Geographic position (lat., long.) | Approximate area at low tide (m^2^) | Average N over all years |
| --- | --- | --- | --- |
| White-1 | N 58° 49' 14.91", E 11° 2' 2.81" | 10 | 1700 |
| White-2 | N 58° 49' 18.99", E 11° 2' 0.49" | 30 | 3600 |
| White-3 | N 58° 49' 31.06", E 11° 2' 29.93" | 30 | 1100 |
| White-4 | N 58° 48' 54.99", E 11° 2' 38.11" | 60 | 280 |
| Banded-1 | N 58° 49' 9.69", E 11° 1' 45.16" | 30 | 670 |
| Banded-2 | N 58° 49' 17.27", E 11° 2' 1.42" | 60 | 1000 |
| Banded-3 | N 58° 49' 43.34", E 11° 3' 25.70" | 180 | 2200 |
| Red | N 58° 49' 22.39", E 11° 1' 42.03" | 50 | 1100 |

Table S2. Model fitted allele and phenotype frequencies in the first sample of snails after transplantation in each skerry population. Models are Directional Selection (**DS**), Heterozygote Advantage (**HA**), Frequency Dependence (**FD**) and migration (**M**). Note that the estimates for White-4 are for 1996, after recovery from a severe bottleneck.

|  | Fitted starting allele frequencies, *p*_0_ | | | | Calculated starting phenotype frequencies | | | |
| --- | --- | --- | --- | --- | --- | --- | --- | --- |
|  | **DS** | **HA** | **FD** | **M** | **DS** | **HA** | **FD** | **M** |
| White-1 | 0.29 | 0.35 | 0.36 | 0.32 | 0.49 | 0.58 | 0.59 | 0.53 |
| White-2 | 0.35 | 0.35 | 0.34 | 0.36 | 0.57 | 0.57 | 0.56 | 0.59 |
| White-3 | 0.28 | 0.28 | 0.27 | 0.29 | 0.48 | 0.48 | 0.47 | 0.49 |
| White-4 | 0.47 | 0.54 | 0.54 | 0.51 | 0.72 | 0.79 | 0.79 | 0.76 |
| Banded-1 | 0.34 | 0.34 | 0.33 | 0.34 | 0.56 | 0.56 | 0.38 | 0.56 |
| Banded-2 | 0.26 | 0.26 | 0.26 | 0.26 | 0.46 | 0.46 | 0.46 | 0.46 |
| Banded-3 | 0.35 | 0.36 | 0.36 | 0.35 | 0.57 | 0.59 | 0.59 | 0.58 |
| Red | 0.29 | 0.34 | 0.33 | 0.31 | 0.50 | 0.56 | 0.56 | 0.53 |

Table S3. Tests of colour heterogeneity within skerry populations. Due to low densities in some years, only one sample was taken from all over the skerry (DD). In 2003, only single samples were taken (not included here). NS – no significant, * P < 0.05 after sequential Bonferroni correction within colour.

Table S3 continued

Table S4. Model log likelihood estimates for all models and skerries (same as in Table 2) but also including pooled estimates.

|  | Model log likelihood (-2LL) estimates | | | |
| --- | --- | --- | --- | --- |
| Colour | Migration | Directional selection | Heterozyg. advantage | Frequency dependent |
| White-1 | 104.3 | 117.7 | 87.8 | **85.6** |
| White-2 | 75.6 | 70.0 | 70.0 | **69.6** |
| White-3 | 73.7 | 72.2 | 72.2 | **71.4** |
| White-4 | 53.9 | 58.5 | 52.4 | **51.7** |
| White pooled-I^1^ | 544.1 | 547.0 | 544.2 | 543.3 |
| White pooled-II^2^ | 370.5 | 353.9 | 353.7 | 354.0 |
| White combined | 307.3 | 318.4 | 282.4 | 278.3 |
| Banded-1 | 99.5 | 98.8 | 98.8 | **98.4** |
| Banded-2 | 80.7 | 80.7 | 80.7 | **78.9** |
| Banded-3 | 86.9 | 87.3 | 86.3 | **86.1** |
| Banded pooled-I^1^ | 301.5 | 301.0 | 300.4 | 300.5 |
| Banded pooled-II^2^ | 272.8 | 273.4 | 272.4 | 273.4 |
| Banded combined | 267.2 | 266.8 | 265.7 | 263.3 |
| Red | 111.6 | 116.8 | **108.0** | 108.6 |
| ^1^Same starting frequency. ^2^Site-specific starting frequency | | | | |

Table S5. Test of deviation from temporal variation caused by drift in frequencies of conspicuous colours in non-target populations (e.g. frequencies of banded phenotype in populations manipulated for the white phenotype). The chi-square test compares the drift model (intercept = 0, slope = 1) with the fitted model and slopes less than 1 suggest balancing selection. Significance after sequential Bonferroni correction (within colour morphs) is indicated (*).

Table S6. Among population variation in frequencies of non-target colours. Variation in frequency among skerries and among years was analysed using a general linear model with quasibinomial error distribution in the lme package in R for the White and Banded skerries and using *χ* ^2^ over years for the single Red skerry. NS – no significant, * P < 0.05, *** P < 0.001.

| Colour | Mean phenotype frequency (%) on manipulated skerries | | | | Variation among skerries | Variation among years |
| --- | --- | --- | --- | --- | --- | --- |
|  | White-1 | White-2 | White-3 | White-4 |  |  |
| Banded | 3.1 | 5 | 2 | 0.4 | F_3,39_ = 13.3*** | NS |
| Red | 0.1 | 1.5 | 0.1 | 0.1 | F_3,39_ = 15.6*** | NS |
|  | Banded-1 | Banded-2 | Banded-3 |  |  |  |
| White | 3.9 | 6.7 | 1 |  | F_3,39_ = 36.7*** | NS |
| Red | 3.8 | 0.6 | 0.1 |  | F_3,39_ = 17.6*** | NS |
|  | Red |  |  |  |  |  |
| White | 7 |  |  |  | - | *χ*^2^_10_ = 22.1* |
| Banded | 2.8 |  |  |  | - | NS |

Table S7. Background frequencies of conspicuous colour morphs from islands and skerries in the study area.

| Sample | Type of location | Red  (%) | White  (%) | Banded  (%) | N | Sampling year |
| --- | --- | --- | --- | --- | --- | --- |
| CZA, Ramsö, 2013 | Island | 0.7 | 5.2 | 1.7 | 290 | 2013 |
| CZE, Ramsö 2015 | Island | 0.0 | 4.5 | 2.1 | 290 | 2015 |
| CZB, Inre Arsklovet | Island | 1.3 | 0.0 | 0.0 | 239 | 2014 |
| CZC, Ramsökalv | Island | 0.0 | 0.7 | 6.9 | 289 | 2014 |
| CZD, Yttre Arsklovet | Island | 0.0 | 0.3 | 2.4 | 290 | 2014 |
| Saltö, exposed rocks^1^ | Island | 3.4 | 23.0 | 3.6 | 414 | 1995 |
| Ursholmen, exposed rocks^1^ | Island | 0.6 | 1.5 | 2.4 | 678 | 1995 |
| Metareskär | Island | 0.0 | 13.5 | 6.0 | 200 | 2000 |
| Flata Kalveskär | Island | 0.5 | 6.5 | 2.0 | 200 | 2000 |
| St Kalvhättan | Island | 0.0 | 1.0 | 0.5 | 200 | 2000 |
| Bergstugan-skerry | Skerry (natural) | 0.0 | 1.5 | 9.0 | 67 | 2003 |
| SO St Sockna-skerry (12) | Skerry (natural) | 0.0 | 44.2 | 0.0 | 43 | 2003 |
| SO St Sockna-skerry (28) | Skerry (natural) | 0.0 | 2.7 | 0.0 | 73 | 2003 |
| Weighted average |  | 0.7 | 6.1 | 2.8 | 3273 |  |
| *Baseline frequencies of non-target morphs in manipulated populations* | | *0.9%* | *4.7%* | *2.7%* |  |  |
| ^1^ Data from Ekendahl & Johannesson, 1997 | |  |  |  |  |  |

Table S8. Comparison between different fits (-2LL) of the frequency dependent model in which the equilibrium frequency was constrained to the observed background frequency (0.048 for the white populations) and the unconstrained model. Chi-squared statistics were used to evaluate the differences in log-likelihood estimates. In none of the cases did the constrained model give a better fit to the data than the unconstrained model.

|  | Constrained | Un-constrained | Constrained vs Unconstrained | df |
| --- | --- | --- | --- | --- |
| White-1 | 94.6 | 85.6 | 9.0** | 1 |
| White-2 | 80.6 | 69.6 | 11.0*** | 1 |
| White-3 | 74.9 | 71.4 | 3.5 | 1 |
| White-4 | 53.8 | 51.7 | 2.1 | 1 |
| White summarized | 303.9 | 278.3 | 25.6*** | 4 |

**P<0.01, ***P<0.001 after sequential Bonferroni correction

Figure S1. Estimated population sizes of skerry populations over the experimental period.

Fig. S2. Colour morphs illustrating introgression of alleles for band from the Swedish wave ecotype *L. saxatilis* (in which the banded phenotype is widespread) to the Swedish crab ecotype where banded phenotypes are absent. The left individual has the banded phenotype (white band) on dark ground colour typical of wave ecotype snails, while the right individual has the typical ground colour of a crab ecotype. The two individuals in the middle are colour phenotypes only found in the hybrid zone and they illustrate how the ground colour of the crab ecotype has combined with the allele for banding from the wave ecotype, even influencing the colour of the band in the left of the two intermediate phenotypes. Photo: Fredrik Pleijel.


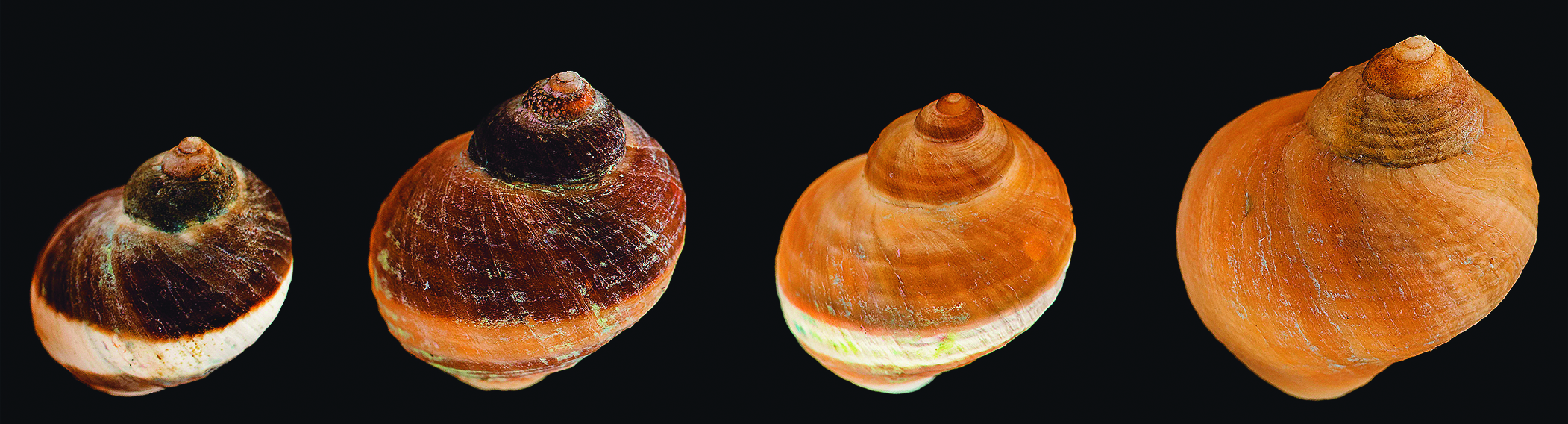

Supplement: Supplementary Information [file hdy201677x1.docx]
